# Dementia in Brazil and the public health response

**DOI:** 10.1002/alz.086497

**Published:** 2025-01-09

**Authors:** Cleusa P Ferri

**Affiliations:** ^1^ Federal University of Sao Paulo (UNIFESP), Sao Paulo Brazil

## Abstract

**Background:**

(1) to present the main findings of two National Dementia Reports developed in partnership with the Brazilian Ministry of Health; (2) to summarize and discuss the proposed public health initiatives to respond to the challenges identified in the reports.

**Methods:**

Desk review, bibliometric study, cross sectional data and secondary analysis of existing data.

**Results:**

There is a lack of knowledge in Brazil in most parts of the country about the epidemiology as well as other important dementia‐related factors. The majority of research conducted so far has been undertaken in the Southeast, and mainly in Sao Paulo, the richest state.

A Delphi consensus was conducted which indicated that there are about 2.5 million people living with dementia in the country, and the report estimated that 80% are not diagnosed. All those diagnosed and treated in the national health system have care needs that, for the most part, are not being met. The costs associated with treatment and care are high, with 73% of the total costs being informal costs, which are mainly borne by the family. Research into dementia has increased, but only at the same pace as health research in general. Moreover, most of the research is related to the diagnosis of the disease and its causes and mechanisms and little has been on epidemiology, care for people with dementia and support for caregivers.

A few states and municipalities have approved, or are in the process of approving, legislation relating to policies, and a national bill has been approved in the Senate, and is in the process of being approved in the Chamber of Deputies. In addition, the Ministry of Health, together with other ministries, such as the Social Development and Education Ministries, has put forward an important initiative regarding long‐term care for chronic conditions.

**Conclusion:**

Although there has been important progress, Brazil does not have yet a national dementia plan and a clear strategy that can increase diagnosis rates and improve care for individuals with dementia, and provide greater support for their carers, while taking into account the geographical, socioeconomic and cultural differences within the country.

